# Cosmesis and body image after single-port laparoscopic or conventional laparoscopic cholecystectomy: *a multicenter double blinded randomised controlled trial (SPOCC-trial)*

**DOI:** 10.1186/1471-2482-11-24

**Published:** 2011-09-12

**Authors:** Daniel C Steinemann, Dimitri A Raptis, Georg Lurje, Christian E Oberkofler, Roland Wyss, Adrian Zehnder, Mickael Lesurtel, René Vonlanthen, Pierre-Alain Clavien, Stefan Breitenstein

**Affiliations:** 1 Department of Surgery, Division of Visceral and Transplantation Surgery, University Hospital Zurich, 8091 Zurich, Switzerland; 2 Department of Surgery, Cantonal Hospital Winterthur, 8401 Winterthur, Switzerland

## Abstract

**Background:**

Emerging attempts have been made to reduce operative trauma and improve cosmetic results of laparoscopic cholecystectomy. There is a trend towards minimizing the number of incisions such as natural transluminal endoscopic surgery (NOTES) and single-port laparoscopic cholecystectomy (SPLC). Many retrospective case series propose excellent cosmesis and reduced pain in SPLC. As the latter has been confirmed in a randomized controlled trial, patient's satisfaction on cosmesis is still controversially debated.

**Methods/Design:**

The SPOCC trial is a prospective, multi-center, double blinded, randomized controlled study comparing SPLC with 4-port conventional laparoscopic cholecystectomy (4PLC) in elective surgery. The hypothesis and primary objective is that patients undergoing SPLC will have a better outcome in cosmesis and body image 12 weeks after surgery. This primary endpoint is assessed using a validated 8-item multiple choice type questionnaire on cosmesis and body image. The secondary endpoint has three entities: the quality of life 12 weeks after surgery assessed by the validated Short-Form-36 Health Survey questionnaire, postoperative pain assessed by a visual analogue scale and the use of analgesics. Operative time, surgeon's experience with SPLC and 4PLC, use of additional ports, conversion to 4PLC or open cholecystectomy, length of stay, costs, time of work as well as intra- and postoperative complications are further aspects of the secondary endpoint. Patients are randomly assigned either to SPLC or to 4PLC. Patients as well as treating physicians, nurses and assessors are blinded until the 7th postoperative day. Sample size calculation performed by estimating a difference of cosmesis of 20% (alpha = 0.05 and beta = 0.90, drop out rate of 10%) resulted in a number of 55 randomized patients per arm.

**Discussion:**

The SPOCC-trial is a prospective, multi-center, double-blind, randomized controlled study to assess cosmesis and body image after SPLC.

**Trial registration:**

(clinicaltrial.gov): NCT 01278472

## Background

Since the first laparoscopic cholecystectomy in 1985, the laparoscopic approach has emerged as the treatment of choice for symptomatic gallstone disease [1,2]. Advantages of laparoscopic compared with open surgery include improved cosmesis, pain control, shorter convalescence and the absence of the formation of intra-abdominal adhesions.

Traditionally laparoscopic cholecystectomy is performed with four ports. In an attempt to further reduce operative trauma and improve cosmetic results following laparoscopic cholecystectomy, there has been a trend toward minimizing the number of incisions and ports required. These new operative techniques are called natural orifice transluminal endoscopic surgery (NOTES) [3,4] or single-port laparoscopic surgery (SPLC) [5] and were developed in order to improve cosmesis and further reduce the invasiveness of conventional laparoscopy [6].

A MEDLINE (Pubmed Central database) search (May 14, 2011) using the keywords 'cholecystectomy' and 'single port', 'single incision', 'single site' or 'single provided 334 articles. However, there were only two randomized controlled studies which were published. These included a low number of patients and focused on postoperative pain [7,8]. Current data show that SPLC is feasible with a low rate of conversion to 4-Port conventional laparoscopic cholecystectomy (4PLC) ranging from 2 to 9.3%. The main reason for conversion was the presence of acute inflammation [9,10]. Systemic reviews showed an incidence of biliary tract injury between 0.09 and 0.7%. [9-11]. However, high evidence regarding safety of SPLC is missing.

SPLC is regarded to provide excellent results in cosmesis with almost no visible scar [12,13]. Yet, the importance of the cosmesis has been challenged as patients also seem to be quite satisfied after 4PLC [14].

Since the available devices for SPLC have been designed to enable an almost scarless procedure the hypothesis of cosmesis improvement has to be proven. No previously published RCT on this topic was designed with the focus on cosmesis. We therefore designed a double-blinded randomized controlled trial on SPLC versus 4PLC (SPOCC-trial) evaluating cosmesis and body image as a primary endpoint.

## Methods/Design

### Study objectives

The SPOCC trial is a prospective, multi-center, double blinded, randomized controlled study comparing Single-Port laparoscopic cholecystectomy with 4-port conventional laparoscopic cholecystectomy in elective surgery for symptomatic cholecystolithiasis. Patients with an indication for an elective cholecystectomy are randomized for either (A) single-port laparoscopic cholecystectomy (SPLC) or (B) conventional 4-port laparoscopic cholecystectomy (4PLC). The hypothesis of this trial is that patients undergoing SPLC will have a better outcome in cosmesis and body image 12 weeks after surgery.

### Endpoints

The primary endpoint of the study concerns patient's satisfaction with cosmesis and body image 12 weeks after surgery. This endpoint is assessed using a validated cosmesis and body image score (CBIS) that was previously used in surgery for Crohn's disease [15] and in donor nephrectomy [16]. This score is calculated on an 8-item multiple-choice type questionnaire (Table 1) ranging between 8 and 48 points.

**Table 1:** Cosmesis and Body image questionnaire consisting of a body image score (items 1 to 5) and a cosmetic score (items 6 to 8)

1	Are you less satisfied with your body since the operation? □ 1 = no, not at all □ 2 = a little bit □ 3 = quite a bit □ 4 = yes, extremely
2	Do you think the operation has damaged your body? □ 1 = no, not at all □ 2 = a little bit □ 3 = quite a bit □ 4 = yes, extremely
3	Do you fell less attractive as a result of your operation? □ 1 = no, not at all □ 2 = a little bit □ 3 = quite a bit □ 4 = yes, extremely
4	Do you feel less feminine/masculine as a result of your operation? □ 1 = no, not at all □ 2 = a little bit □ 3 = quite a bit □ 4 = yes, extremely
5	Is it difficult to look at yourself naked? □ 1 = no, not at all □ 2 = a little bit □ 3 = quite a bit □ 4 = yes, extremely
6	On a scale from 1 to 7, how satisfied are you with your scar(s)? □ 1 = very unsatisfied □ 2 = quite unsatisfied □ 3 = a bit unsatisfied □ 4 = not unsatisfied/not satisfied □ 5 = a bit satisfied □ 6 = quite satisfied □ 7 = very satisfied
7	On a scale from 1 to 7, how would you describe your scar(s)? □ 1 = revolting □ 2 = quite revolting □ 3 = a bit revolting □ 4 = not revolting/not beautiful □ 5 = a bit beautiful □ 6 = quite beautiful □ 7 = very beautiful
8	Could you score your own scar(s) on a scale from 1 to 10? (1 = ugliest scar imaginable, 10 = almost scarless)

Scores from 8 to 48.

The secondary endpoint is a combination of several variables: the quality of life 12 weeks after surgery assessed by the validated Short-Form-36 Health Survey questionnaire [17], postoperative pain assessed by a visual analogue scale daily assessment and the use of analgesics. Operative time, use of additional ports, conversion to open cholecystectomy, length of stay, costs, elapsed time before going back to work as well as intra- and postoperative complications represent aspects of the secondary endpoint. The postoperative complications are classified according to the Clavien-Dindo system [18].

### Power of the study

A clinically relevant improvement of the cosmesis and body image score (CBIS) [15] is defined as an improvement of 20% of the cosmesis score (8 points). Given the reported standard deviation of the CBIS between 4-6 [19] and using (alpha = 0.05 and beta = 0.90), two groups of 49 patients are needed. This is based on a two-sided significance level (alpha) of 0.05 and a power of 0.90. Estimating a 10% dropout rate, which is common in randomized controlled trials [20], 55 patients will be randomized per arm.

### Inclusion criteria

Inclusion criteria are

- elective cholecystectomy

- 18 years of age

- ability to understand the study purpose (German language)

- written informed consent

### Exclusion criteria

Exclusion criteria are

- pregnant women

- proven or suspected liver cirrhosis

- coagulopathy (platelet count below 50'000/μl)

- double medication on platelet antagonists (acetylsalicylic acid and clopidogrel)

- international normalized ratio (INR) below 1.4

- Non-German speakers

- patients who are unable to understand the study purpose.

- family members of study investigators or employees of the participating centres

### Participating surgeons and clinics

To prevent surgeon bias, the requirements to qualify as a surgeon are equal in both arms. Since the present study represents a so-called pragmatic randomised control trial [21], the study should reflect daily practice for cholecystectomy. Operating surgeons are senior residents, chief residents, attending surgeons as well as consultant surgeons. The operating surgeon will need a minimal experience of 35 4PLC cholecystectomies,10 SPLC and must participate in a one-day workshop for SPLC.

Participating centres are the Division of Visceral and Transplantation Surgery of the University Hospital Zurich and the Department of Surgery of the Cantonal Hospital in Winterthur, Switzerland.

### Randomization

Patients have to sign an informed consent in the outpatient clinic. Randomization is performed online (internet randomization module http://www.randomizer.at[22] at the time of transfer to the operating room.

### Double blinding

Patients as well as surgeons, treating physicians and nurses are blinded until the 7^th ^postoperative day. At the end of the operation an opaque wound dressing will be applied at the four port-sites for 4PLC in both treatment arms. The dressings are occlusive and water-resistant. The skin will be closed with subcutaneous absorbable stitches. Additionally the wound will be sealed using fibrin glue to avoid any blood spots on the dressing. Each dressing will be marked with a sterile marker so that any violation of the blinding will be noticed and documented. Data collection as well as clinical controls of patients is performed by investigative independent physicians.

Any change of dressings before 7 days postoperatively are documented.

### Data collection and statistics

**Figure 1:**
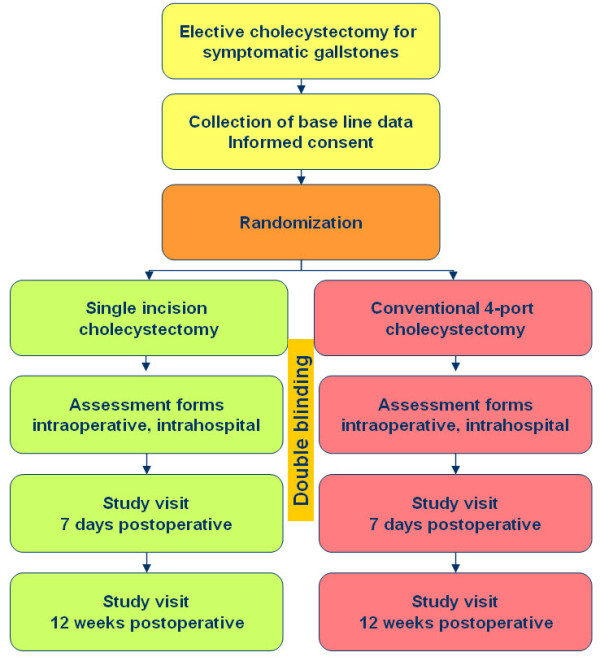
**Study flowchart**.

Informed consent of patients is performed in the outpatient clinic. Single port cholecystectomy is not offered to patients outside of the study. Data including results of questionnaires is collected on an internet-based secured study information platform [23]. The CBIS questionnaire is filled out at the outpatient clinic preoperatively as well as on the day of admission, 1 and 12 weeks postoperatively. The SF-36 quality of life questionnaire is filled out preoperatively in the outpatient clinic and 12 weeks postoperatively. Pain variables are assessed on a daily basis during hospital stay (Figure 1). Data analysis is performed in accordance with the intention-to-treat principle. Groups are compared using an Independent Samples T-test, Wilcoxon test, or Chi-square test.

### Ethics

This study is conducted in accordance with the principles of the Declaration of Helsinki and 'good clinical practice' guidelines. The principal investigator has completed a course in 'good clinical practice' certified by the Swiss Agency for Therapeutic Products (Swissmedic). The independent medical ethics committee of canton Zurich (Kantonale Ethikkommission Zürich, Switzerland) has approved the study protocol. This trial was registered at clinicaltrial.gov on January 14, 2011 (trial number: NCT01278472).

### Surgical technique

Patients are placed in the French position with the surgeon between the legs, the cameraman (first assistant) on the left of the patient, the second assistant (facultative) on the right, the nurse near the right hand of the surgeon, and the monitor shoulder level to the patient. The endoscopic equipment in terms of optic, monitor, gas supply, suction device, graspers, monopolar hook and endobag are equal in both groups. For surgical disinfection of the skin iodopovidone is used.

#### Single-Port laparoscopic cholecystectomy (SPLC)

**Figure 2:**
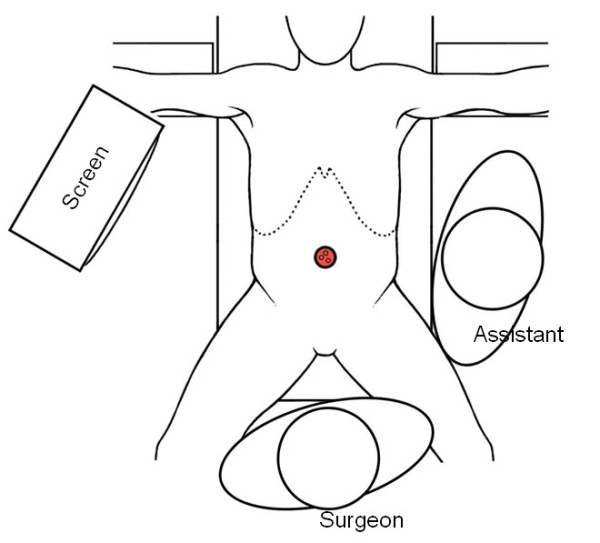
**Setting in Single-Port laparoscopic cholecystectomy**.

A transumbilical straight 20 to 25 mm skin and fascia incision is performed (Figure 2). The peritoneum is opened and a SILS™ PT12 Port (Covidien Inc., Norwalk, California, USA) is introduced. This port obtains four openings: one for gas insufflation and three that can accommodate trocars ranging from sizes 5 to 12 mm. The compressibility of the elastic polymer allows for the access ports to expand and form fit the space in which it resides as well enabling the ports to pass through the working channels. The pneumoperitoneum is maintained at 10-12 mmHg. A 5-mm 45° long scope (HD EndoEye™, Olympus Europa Holding GmbH, Hamburg, Germany) is introduced through one of the openings in the SILS™ Port. The patient is then placed in an anti-Trendelenburg position. The fundus of the gallbladder is grasped and the EndoGrab™ (Virtual Ports Ltd., Caesarea, Israel) internally anchored retraction system is introduced and placed at the fundus and the peritoneum parietal. The infundibulum is laterally retracted using a bending grasper (Endograsp roticulator™, Covidien Inc., Norwalk, California, USA). Subsequently good exposure of the triangle of Callot is achieved. The dissection is done using a monopolar dissection hook. The cystic artery and duct are first dissected and then separately clipped with a standard 5 mm clip applicator (Covidien, Norwalk, California, USA). The gallbladder is pushed upright and dissected free from the liver by means of the monopolar hook. Once the gallbladder is free from the adjacent tissues, the 5 mm trocar is exchanged for a 10 mm trocar which is inserted through the SILS™ access device. An Endocatch bag (Endocatch Gold, 10 mm; Covidien Inc., Norwalk, California, USA) is inserted and the gallbladder is extracted. The umbilical fascia is closed using absorbable Vicryl suture, and the natural scar of the umbilicus is restored using intracutaneous stitches and finally covered by dermal glue (Dermabond™; Ethicon Endosurgery Inc., Smithfield, RI, USA). The SPLC technique which was used, was presented as a video at the 98^th ^congress of the Swiss Society of Surgery [24].

#### 4-Port laparoscopic cholecystectomy

**Figure 3:**
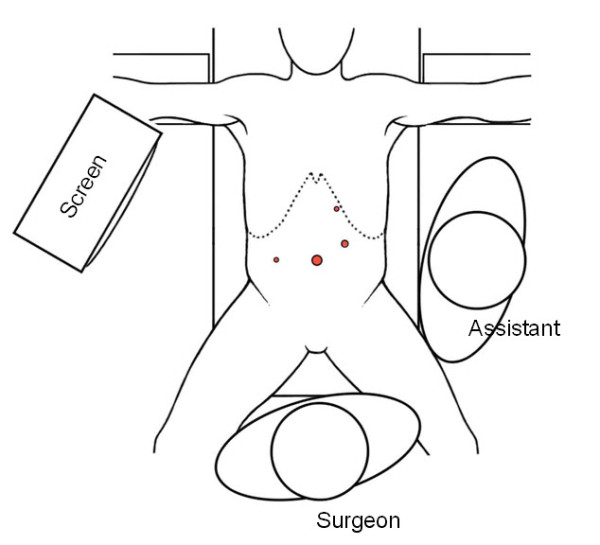
**Setting in 4-Port laparoscopic cholecystectomy**.

A 12 mm paraumbilical skin incision is performed, the fascia and the peritoneum are incised in an open approach and a 12 mm Hasson-type blunt trocar (Ethicon Endosurgery Inc., Smithfield, RI, USA) is introduced. The pneumoperitoneum is maintained at 10-12 mmHg. A 10 mm 45° long scope (HD EndoEye™, Olympus Europa Holding GmbH, Hamburg, Germany) is introduced. Now additional 5 mm trocars are placed under optical control subxyphoidal on the left side of the falciforme ligament and in the right lateral upper abdomen. These trocars are used as endograspers to retain the fundus and the infundibulum of the gallbladder. A further 10 mm reusable trocar is placed in the left middle abdomen. This trocar is used as dissection instruments, swabs and the endobag (Figure 3). Optimal exposure of the triangle of Callot is achieved. The cystic artery and duct are first dissected and then separately clipped with a standard 5 mm clip applicator (Covidien, Norwalk, California, USA). The gallbladder is pushed upright and dissected free from the liver by means of the monopolar hook. Once the gallbladder is free from the adjacent tissues a standard Endocatch bag (Endocatch Gold, 10 mm; Covidien, Norwalk, CT, USA) is inserted and the gallbladder is extracted. The paraumbilical fascia und the fascia in the left middle abdomen is closed using absorbable Vicryl suture. The skin is closed using intracutaneous stitches and covered by dermal glue (Dermabond; Ethicon Endosurgery, Inc., Smithfield, RI, USA).

## Discussion

The prevalence of diagnosed cholecystolithiasis is high: 14.4% in men and 25.3% in women. About half of these patients are symptomatic and therefore candidates for cholecystectomy [25]. Laparoscopic cholecystectomy has become one of the most frequently performed procedures in visceral surgery. Current efforts focus on minimizing the operative trauma and improving cosmesis by reduction of trocars. On the other hand innovations in this field represent a significant economic burden. Hence, the postulated advantages for the patients of SPLC as well as cost effectiveness need to be evaluated in prospective randomized studies.

While many retrospective case series on SPLC are available [9,10], only two RCTs have been published showing reduced postoperative pain [8,26].

Currently 9 RCTs are registered in the international trial database (clinicaltrials.gov) (Table 2). Two trials have small sample sizes and focus on postoperative pain (NCT01348620, NCT01094379). The majority of trials focus on postoperative pain or costs. One trial with the most extensive sample size (600-patients) investigates differences in morbidity (Trial of the European Association of Endoscopic Surgery, EAES, NCT01104727).

**Table 2:** Listed recruiting Randomized Controlled Trials (RCT) on clinicaltrial.gov (search date: May-14, 2011). QoL = Quality of life

trial number	study Center	enrollment	1° endpoint	2° endpoint(s)	masking
NCT01348620	Sacro Cuore Don Calabria	58	Pain	Cosmesis (VAS)	Double blind
NCT01094379	Athens	40	Pain	Cosmesis (1 month), OR time, QoL	Single blind
NCT01104727	EAES	600	Morbidity rate	Pain, cosmesis (2 months)	Open label
NCT00974194	Lausanne	260	Pain	Complications, costs, cosmesis (1, 3, 12 months), complications	Double blind
NCT01268748	Copenhagen	120	Pain	Cosmesis (1 month), well-being score	Double blind
NCT01195285	Saint Luke's Health System	150	Costs	Pain, morbidity, QoL	Double blind
NCT00904865	Geneva	200	Cosmesis, body image and QoL (1 month)	Morbidity, Pain, OR time	Open label
NCT01339325	San Giovanni Addolorata	180	Compound of length of stay, pain, cosmesis and QoL	OR time, conversion rate, complication rate	Open label
NCT01278472	Zurich	110	Cosmesis (3 months)	Pain, QoL, conversion rate, convalescence	Double blind

Cosmesis and body image (after 1 month) as a primary endpoint is considered in only one registered trial (NCT00904865) while another study focuses on a combined primary endpoint consisting of quality of life assessed by SF-36, cosmesis and pain (NCT01339325-trial) (Table 2).

The present SPOCC-trial has two specific advantages, such as the time point of cosmesis assessment 3 months after surgery, which represents a reasonable follow-up time point, using a validated questionnaire and scoring system [15]. The second strength of the trial design is the double blinding.

## Competing interests

The authors declare that they have no competing interests.

## Authors' contributions

DCS and SB drafted the manuscript. DCS, GL, CEO and SB designed the protocol and co-authored the writing of the manuscript. DAR contributed to the design of the protocol and calculation of the power of the study. All other authors participated in the design of the study and are local investigators in the participating centers. All authors were involved in editing the manuscript and approved the final text of the manuscript.

## Pre-publication history

The pre-publication history for this paper can be accessed here:

http://www.biomedcentral.com/1471-2482/11/24/prepub
